# MXene/Cellulose Composite Cloth for Integrated Functions (*if*-Cloth) in Personal Heating and Steam Generation

**DOI:** 10.1007/s42765-023-00345-w

**Published:** 2023-12-22

**Authors:** Jian Chang, Bo Pang, Hao Zhang, Kanglei Pang, Miao Zhang, Jiayin Yuan

**Affiliations:** Department of Materials and Environmental Chemistry, Stockholm University, 10691 Stockholm, Sweden

**Keywords:** Composite cloth, Solar heating, Personal heating, Steam generation

## Abstract

Given the abundant solar light available on our planet, it is promising to develop an advanced fabric capable of simultaneously providing personal thermal management and facilitating clean water production in an energy-efficient manner. In this study, we present the fabrication of a photothermally active, biodegradable composite cloth composed of titanium carbide MXene and cellulose, achieved through an electrospinning method. This composite cloth exhibits favorable attributes, including chemical stability, mechanical performance, structural flexibility, and wettability. Notably, our 0.1-mm-thick composite cloth (RC/MXene IV) raises the temperature of simulated skin by 5.6 °C when compared to a commercially available cotton cloth, which is five times thicker under identical ambient conditions. Remarkably, the composite cloth (RC/MXene V) demonstrates heightened solar light capture efficiency (87.7%) when in a wet state instead of a dry state. Consequently, this cloth functions exceptionally well as a high-performance steam generator, boasting a superior water evaporation rate of 1.34 kg m^−2^ h^−1^ under one-sun irradiation (equivalent to 1000 W m^−2^). Moreover, it maintains its performance excellence in solar desalination processes. The multifunctionality of these cloths opens doors to a diverse array of outdoor applications, including solar-driven water evaporation and personal heating, thereby enriching the scope of integrated functionalities for textiles.

**Graphical Abstract:**

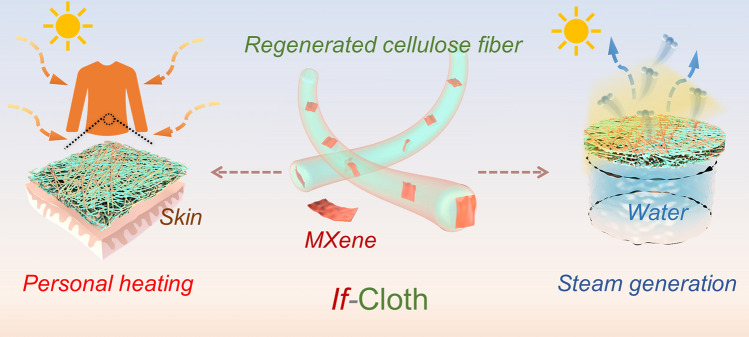

**Supplementary Information:**

The online version contains supplementary material available at 10.1007/s42765-023-00345-w.

## Introduction

The conversion and utilization of solar energy globally are widely recognized as pivotal solutions for mitigating the ongoing energy crisis and addressing pressing environmental concerns. Among the various pathways for energy conversion, the conversion of light into heat stands out as a conceptually straightforward and practically convenient approach. The heat generated through this process finds versatile applications in water purification and desalination [1–5], the development of personal heating textiles [6–9], bactericidal treatments [10, 11], catalytic conversions [12, 13], actuation mechanisms [14], deicing processes [15], and more. Over the past decade, an array of photothermal materials has emerged, exhibiting broad-spectrum absorption spanning the entire solar spectrum and showcasing high efficiency in converting solar energy to heat [16, 17]. However, the complexities of achieving an optimal balance between factors such as efficiency, cost, scalability, stability, and sustainability pose challenges to their potential commercialization. Consequently, there is a growing emphasis on achieving multifunctional integration to enhance adaptability across a broader spectrum of real-life applications.

2D titanium carbide (Ti_3_C_2_T_x_), known as MXene, has garnered attention for its remarkable ability to efficiently absorb solar energy through excellent light-to-heat conversion capabilities. Beyond this, MXenes exhibit low cytotoxicity, favorable mechanical properties, high hydrophilicity, and fire retardancy, rendering them versatile candidates for various solar-related applications [18–26]. However, despite these merits, a notable drawback of MXene-based materials is their susceptibility to degradation when exposed to prolonged air, moisture, and light periods. Concurrently, the structural integrity of MXene-decorated materials is susceptible to damage from external mechanical forces (such as abrasion and washing), particularly when MXene is applied to substrate surfaces through weak interactions. These dual challenges present significant obstacles to the practical implementation of MXene-based materials.

To improve the stability of photothermal materials, chemically stable, mechanically robust, and photostable polymers (e.g., polyamide, polystyrene, and polyacrylonitrile) are employed as matrix to encapsulate solar absorbents, instead of commonly decorating them onto matrix surface [27]. In demonstrating this concept, the present study utilized a biodegradable polymer, regenerated cellulose (RC), to encapsulate inorganic MXene nanosheets within a fibrous matrix via an electrospinning technique. The composite photothermal materials, presented as nonwoven cloths, exhibit exceptional absorption across the full solar spectrum (74.7%). Moreover, these materials enhance wettability, structural flexibility, and efficiency of photothermal conversion.

Significantly, the resultant integrated functional cloth (referred to as *if*-Cloth) delivers substantial advantages in practical applications such as solar-driven water evaporation and personal heating. Consequently, the *if*-Cloth achieves a noteworthy water evaporation rate of 1.34 kg m^−2^ h^−1^ and demonstrates a solar-driven water evaporation efficiency of approximately 89.6% under one-sun irradiation. Furthermore, the *if*-Cloth exhibits remarkable personal heating capabilities and excels as wearable attire due to its mechanical and chemical stability, breathability, and rapid water uptake. These attributes position it as a captivating contender for personal thermal management. Experimental tests reveal that a 0.1-mm-thick *if*-Cloth rapidly attains a temperature 5.6 °C higher than its 0.5-mm-thick woven cotton counterpart when placed on simulated skin under identical ambient conditions. The design of *if*-Cloth provides a valuable reference for the utilization of photothermal materials toward diverse practical applications.

## Experimental Section

### Materials

Cellulose acetate (CA, *M*_*n*_ ~ 30 kDa), lithium fluoride (LiF, ≥ 99.98% trace metals basis), N,N-dimethylformamide (DMF, anhydrous, 99.8% purity), ethanol (99% purity), sodium hydroxide (NaOH), and acetone were procured from Sigma-Aldrich. Ti_3_AlC_2_ MAX phase powder (400 mesh) with high purity (≥ 99%) was acquired from Laizhou Kai Ceramics Materials Co., Ltd. Hydrochloric acid (HCl, 37% concentration) was provided by VWR International. Commercial cotton, fleece, and lyocell were purchased from H&M, a clothing company based in Sweden. All remaining chemicals were used as received without requiring further purification.

### Synthesis of Delaminated Ti_3_C_2_T_x_ (MXene)

An MXene dispersion was synthesized following a method akin to the minimally intensive layer delamination (MILD) process, as previously documented [28]. In detail, 1.5 g of high-purity Ti_3_AlC_2_ (MAX) powder was gradually introduced into a mixture of 30 mL of 9 M HCl and 2.4 g of LiF, all while maintaining continuous stirring at 38 °C for 48 h. Subsequently, the resulting multilayer Ti_3_C_2_T_x_ and unetched MAX particles underwent multiple washes using deionized (DI) water. This was followed by a repetitive cycle of agitation and centrifugation (at 11,000 rpm for 1 h per cycle), which continued until the supernatant reached a pH value of 6. Ultimately, a suspension of Ti_3_C_2_T_x_ nanosheets was obtained by exfoliating the resultant slurry via ultrasonication for 1 h under the protection of N_2_ gas and ice.

### The Preparation of CA and CA/MXene Electrospun Membrane

Pristine CA and MXene/CA nanofibers were fabricated using electrospinning. While stirring, a solution consisting of 20 wt% CA was prepared by adding CA powder into a mixture of DMF and acetone (4:6 v/v). This solution was then loaded into a 5 mL syringe for electrospinning. The electrospinning parameters for the CA nanofibers were an applied voltage of 20 kV, a spinning distance of 15 cm between the syringe tip and the aluminum foil substrate, and an injection rate of 0.5 mL h^−1^. The ambient temperature was maintained at 22 °C with a humidity of 40%. The formed nanofibers were dried in a vacuum oven for 12 h at 60 °C to eliminate solvent residues. The thickness of the electrospun nanofibers could be controlled by adjusting the electrospinning time.

In addition to the above, varying amounts of exfoliated MXene nanosheets were uniformly dispersed in the CA solutions while continuously stirring for 12 h. The MXene content embedded within the nanofibers was quantified at 1.3 wt%, 1.9 wt%, 3.3 wt%, 5.7 wt%, and 8.0 wt% relative to CA, as measured using a thermogravimetric analyzer (TGA). The dispersion exhibited sufficient stability to prepare electrospun nanofibers under the same electrospinning conditions for crafting the pristine CA nanofibers. All nanofibers were produced using a lab-scale electrospinning setup.

### Deacetylation of CA and CA/MXene Electrospun Membrane

The electrospun membrane underwent alkaline hydrolysis to eliminate the acetyl groups present in the precursor CA, facilitating the generation of regenerated cellulose nanofibers [29]. Both the CA and CA/MXene electrospun membranes were subjected to deacetylation using a 0.05 M NaOH/ethanol solution at room temperature for 48 h. Following this, the fiber membrane was meticulously rinsed with deionized water to eliminate any remaining traces of NaOH and ethanol from the scaffolds. Ultimately, the membranes were dried in an oven overnight at 40 °C.

### Characterization

Transmission and reflectance spectra of both the electrospun nanofibers and the MXene film were assessed utilizing a UV–Vis spectrophotometer (Agilent Cary 5000 UV–vis-NIR) across the wavelength range of 300–2500 nm. An integrating sphere attachment equipped with a specialized fluorine-based polymer (PTFE) served as a reference for the measurement of reflectance spectra. Determining light absorption involved subtracting the cumulative effects of light transmission and reflection from the incident light total [6, 8].

The phase compositions of the MXene were ascertained through X-ray diffraction analysis (XRD, Malvern Panalytical, Malvern, UK) employing non-monochromatic Cu Kα radiation. Each analysis adopted an acceleration voltage of 40 kV, a current of 40 mA, and a step size of 0.033°. Data collection spanned from 5° to 80°. The surface composition of the samples and the binding energy of the MXene were investigated using X-ray photoelectron spectroscopy (XPS, Thermo Escalab 250XI) under ultrahigh vacuum conditions, maintaining a pressure of around 10^–10^ mbar. A monochromatic Al Kα X-ray source (*hυ* = 1486.6 eV) operated at 150 W was employed.

Scanning electron microscopy (SEM) was conducted on a JSM-7000F microscope (Tokyo, Japan). Atomic force microscopy (AFM, Veeco Instruments, CA) was performed using a Nanoscope V in tapping mode. The water contact angles of the nanofibers were measured using a contact angle goniometer (DSA 25E), employing a water droplet volume of 4 μL.

Thermogravimetric analysis of the nanofibers was executed using a TGA (Discovery TG) under a dry nitrogen atmosphere. The relative mass losses of the nanofibers and MXene were recorded as the temperature was increased from 25 to 600 °C, employing a heating rate of 10 °C/min.

### Solar Heating Measurement

To assess sample temperatures, an IR camera (Testo 872) was utilized for capturing thermal images, while temperature changes were concurrently recorded using a K-type thermocouple (KAIPUSEN) in an air environment. Solar irradiation was administered via a solar simulator (MiniSol LED Solar Simulator, Newport). The simulated sunlight was directed perpendicular to the surface of the samples.

For the outdoor photothermal examination, the testing nanofibers and cotton cloth were positioned on the surface of the simulated skin (adorned with insulating tape possessing an emissivity similar to human skin). This configuration was sealed using heat-resistant tape, and a thermocouple was inserted between the simulated skin and the measured samples. The simulated skin was mounted on a PS foam to provide heat insulation and exposed to sunlight. These outdoor measurements took place in Stockholm, Sweden. Solar irradiance was quantified using a data-logging solar power meter (ISM 410, RS Components Ltd.), while wind speed and ambient temperature were continuously monitored using a flow anemometer (AVM-09, RS Components Ltd.).

### Water Vapor Transmission Rate Test

A plastic cup with a diameter of 3.9 cm was filled with 30 mL of deionized water, and its opening was subsequently sealed using either nanofibers or cotton cloth. This prepared cup was then introduced into an environmental chamber set at 35 °C and a relative humidity of 30%. The mass of water that underwent evaporation was measured by periodically weighing the plastic cup on an electronic balance. Subsequently, the water vapor transmission rate was computed based on the recorded loss in the mass of water from the plastic cup.

### Wicking Test

A pipette wetted a glass platform with 0.1 mL of deionized water. Subsequently, 5 × 5 cm^2^ samples were positioned on the moistened area, and the duration taken for the water to propagate to a circular region with a radius of 1.5 cm on the sample surface was noted. The wicking rate was determined by dividing the wicking area by the wicking time.

### Mechanical Test

Tensile testing was conducted using an Instron 5960 universal testing machine (Instron, USA) equipped with a 100 N load cell. All nanofibrous samples were cut into 3 mm × 30 mm strips, and their thicknesses were measured using a micrometer. These strips were then tested at a constant speed of 5 mm min^−1^ with a gauge length of 10 mm. In this context, tensile stress was defined as the ratio of force to the initial cross-sectional area, while tensile strain represented the ratio of the change in length to the original gauge length. Young’s modulus was calculated from the slope of the linear region on the stress–strain curves obtained during testing. Additionally, toughness was determined by integrating the areas under the stress–strain curves. Each sample was tested five times to ensure statistical reliability, and the mean value was computed.

## Results and Discussion

### Preparation and Characterization of *if*-Cloth

**Fig. 1:**
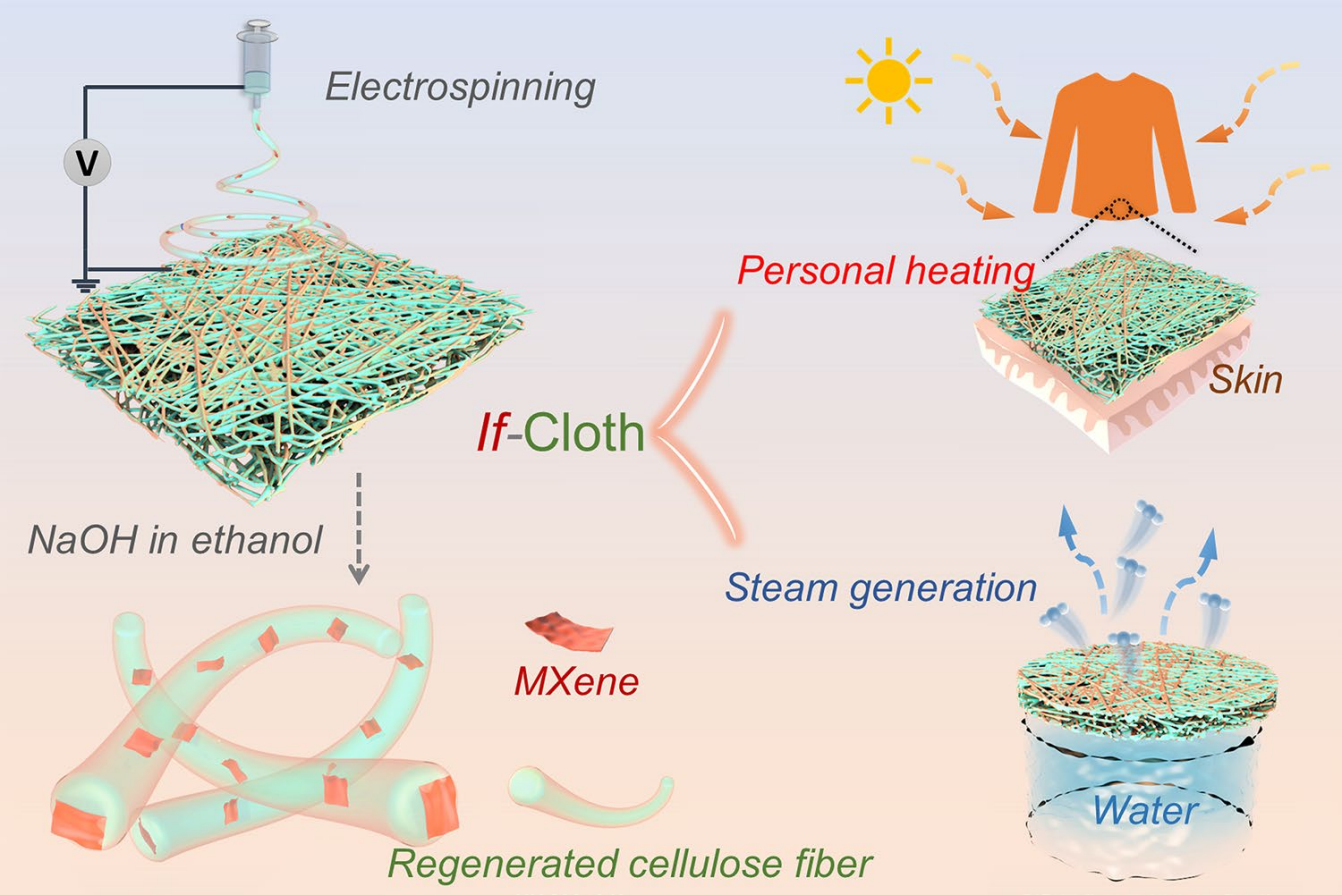
Schematic of the electrospinning method for preparation of composite nanofibers and the practical use of the integrated functional cloth (*if*-Cloth) in personal heating and steam generation

In pursuit of scalability, a direct and budget-friendly fabrication technique known as electrospinning was selected for the mass production of a composite photothermal cloth (Fig. 1) [30]. This approach facilitates the consistent integration of solar-absorbent material within a non-solar absorbing polymeric matrix.

**Fig. 2:**
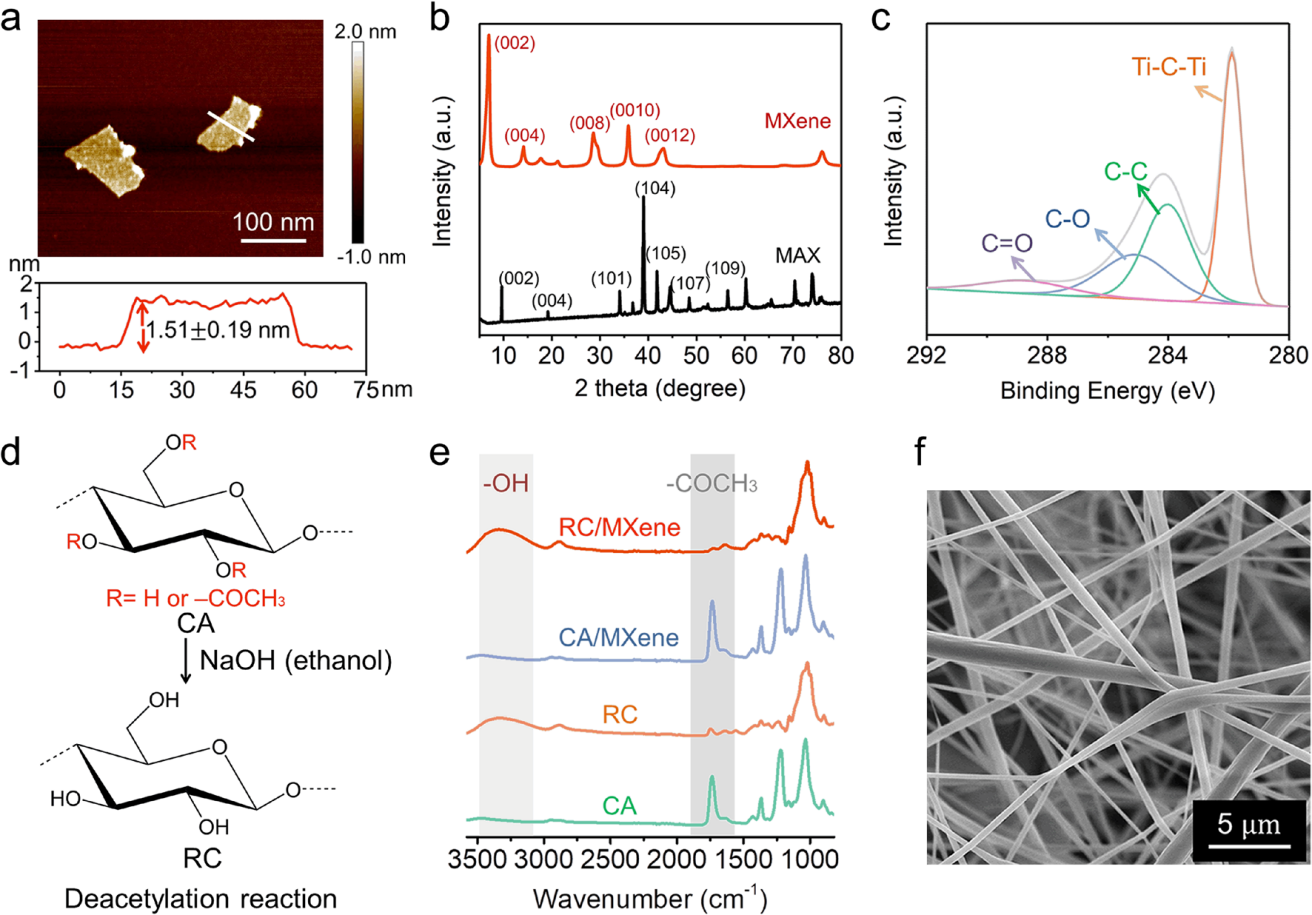
Characterization of MXene and composite nanofibers. **a** AFM image of MXene nanosheets and the corresponding height profile. **b** XRD patterns of the MAX and as-synthesized MXene nanosheets. **c** Fitted XPS spectra of C 1s of MXene nanosheets. **d** Schematic of the deacetylation reaction from CA to RC. **e** FT-IR spectra of CA, RC, CA/MXene, and RC/MXene nanofibers. **f** SEM image of RC/MXene composite nanofibers (RC/MXene IV)

In this design, MXene nanosheets function as the solar absorbent due to their ability to effectively capture the solar spectrum, particularly within the visible light range (Fig. S1). This capability stems from their satisfactory electromagnetic wave absorption capacity and a localized surface plasmon resonance effect [11, 19, 31]. The synthesis of MXene nanosheets followed a well-established procedure [28, 32]. In this process, the aluminum atom layer within the bulk MAX (Ti_3_AlC_2_) was selectively etched away using a LiF/HCl solution, simultaneously allowing for the intercalation of Li^+^ ions into the layered structure. Consequently, the MAX powder's original densely packed crystalline structure transformed into the layered structure characteristic of MXene (Fig. S2). After subjecting the material to sonication treatment, individual-layer MXene nanosheets were obtained. These nanosheets exhibited a lateral size of 0.18 ± 0.07 μm and a thickness of 1.51 ± 0.19 nm, as determined through SEM (Fig. S3) and AFM (Fig. 2a), respectively.

Successful etching and exfoliation were further confirmed by the X-ray diffraction (XRD) patterns (Fig. 2b). The characteristic peaks corresponding to the (101), (104), and (105) planes of the MAX phase at 34.1°, 39.1°, and 41.8°, respectively, disappeared after the etching process [33, 34]. Correspondingly, oxygen (O), hydroxyl (OH) groups (from H_2_O), and fluorine (F) (from LiF) became bound to the titanium (Ti) sites instead of the removed aluminum (Al). This resulted in MXene nanosheets with a uniform distribution of titanium (Ti), carbon (C), oxygen (O), and fluorine (F) elements. This distribution was verified through energy-dispersive X-ray spectroscopy (EDS) elemental mapping results (Fig. S4 and Table S1) and XPS surveys (Fig. 2c and Fig. S5). Analysis suggests that the C 1s spectra of MXene nanosheets could be fitted with four components corresponding to Ti–C–Ti (281.9 eV), C–C (284.1 eV), C–O (285.2 eV), and C=O (288.9 eV) [35, 36].

Cellulose acetate (CA), as one of the most crucial cellulose derivatives being biodegradable and biocompatible [37], can be more easily electrospun into nanofibers than natural cellulose [38]. As such, it was chosen as the initial porous matrix for the photothermal nanofibers. Subsequently, the electrospun CA-based nanofibers transformed regenerated cellulose (RC) by stripping the acetyl groups via alkaline hydrolysis in a NaOH/ethanol solution (Fig. 2d) [39–41].

Comparing the FT-IR spectra of CA, RC, and the composite nanofibers in Fig. 2e, a pronounced band at 1733 cm^−1^, attributed to the C=O stretching vibration of the acetate group, was evident in the spectra of both CA and CA/MXene composite nanofibers [42]. However, this band nearly disappeared after the deacetylation process. Meanwhile, a hydroxyl band spanning 3030–3550 cm^−1^ emerged in RC and RC/MXene composite nanofibers, indicating the successful conversion of CA into RC [29]. This hydrolysis reaction endowed the RC-based nanofibers with improved mechanical strength, heightened surface hydrophilicity, and enhanced chemical resistance against organic and aqueous solutions across a broad pH range from 3 to 12 (Fig. S6).

By encapsulating varying quantities of MXene nanosheets, specifically at 1.3 wt%, 1.9 wt%, 3.3 wt%, 5.7 wt%, and 8.0 wt% (Supplementary Note S1, Fig. S7, and Table S2), the resulting RC/MXene composite nanofibers exhibited a consistent diameter of 0.25 ± 0.07 μm (Fig. 2f, Fig. S8 and Table S3). These nanofibers are labeled RC/MXene I/II/III/IV/V hereinafter. They were randomly stacked to build up nonwoven cloths of ~ 0.1 mm in thinness without detectable transmission of solar light through the nanofibers (Fig. S9).

### Core Performances

**Fig. 3:**
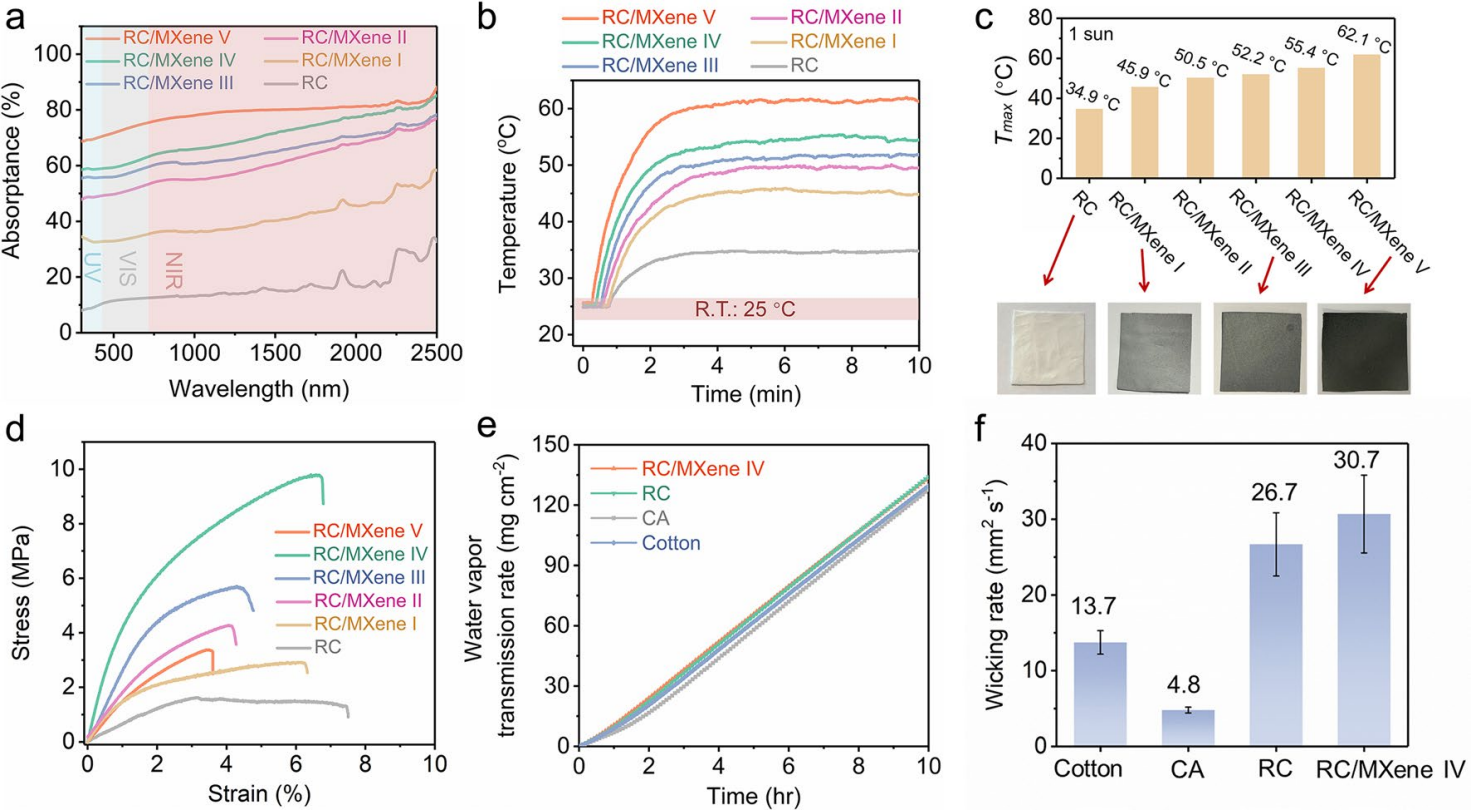
Core performances of *if*-Cloth. **a** UV–Vis–NIR absorption spectra of RC and RC/MXene I/II/III/IV/V nanofibers. **b** Time course plots of their temperatures under one-sun irradiation (1000 W m^−2^). **c** *T*_max_ of RC and RC/MXene I/II/III/IV/V nanofibers and digital images of RC, RC/MXene I, RC/MXene III and RC/MXene V nanofibers. **d** Plots of tensile stress–strain of RC and RC/MXene I/II/III/IV/V nanofibers. Water vapor transmission rate (**e**) and wicking rate (**f**) of RC/MXene IV, RC, CA and cotton cloths

Benefiting from the photothermal capabilities of MXene nanosheets, the absorptivity of RC/MXene composite nanofibers can be augmented by increasing the MXene content within the RC matrix across the entire spectrum of UV, visible, and NIR light (300–2500 nm) (Fig. 3a and Fig. S10). Accordingly, following a 2-min exposure to one-sun irradiation (1000 W m^−2^), the temperatures of the composite nanofibers rapidly escalate from an initial room temperature of 25.0 °C to their peak temperature (referred to as *T*_max_) of 45.9 °C, 50.5 °C, 52.2 °C, 55.4 °C, and 62.1 °C for RC/MXene I/II/III/IV/V, respectively (Fig. 3b, c). In contrast, the *T*_max_ of the pristine RC cloth, with its white surface, only reaches 34.9 °C under identical conditions. MXene nanosheets into nanofibers leads to a darker surface giving a higher surface temperature (Fig. 3c). Notably, RC/MXene V exhibits the most desirable photothermal performance with a solar absorption (termed as *A*_solar_) of 74.7% (Supplementary Note S2) and a *T*_max_ of 62.1 °C, both of which are comparable to the polymer-free MXene film prepared via vacuum filtration of a MXene dispersion (*A*_solar_ ~ 68.6% and *T*_max_ ~ 62.6 °C), as shown in Fig. S11 and Table S4. This suggests that the composite nanofibers, harboring embedded MXene nanosheets, can achieve a photothermal effect akin to that of the pure MXene film. Unlike the pure MXene film, these composite nanofibers exhibit enduring stability in harsh environments (Figs. S12 and S13).

Furthermore, incorporating an appropriately balanced proportion of MXene nanosheets into RC nanofibers significantly enhances the mechanical performance of the bulk fibers, as depicted in Fig. 3d. Notably, the RC/MXene IV cloth exhibits a peak tensile stress of 8.7 ± 2.0 MPa, which is nearly six times that of the MXene-free RC cloth (1.5 ± 0.3 MPa) (Table S5). This enhancement in mechanical properties stems from the effective transmission of stress from the nanofillers to the composite matrix during the deformation process [43]. The alignment of MXene nanosheets can contribute to heightened tensile strength in individual fibers, thus bolstering the overall tensile strength of the fibrous membrane. Additionally, the polar terminal groups (–F, –O, and –OH) present on MXene can establish intermolecular hydrogen bonds with the abundant –OH groups on regenerated cellulose, potentially fortifying the entire composite structure [37]. However, excessive load of MXene nanosheets into RC nanofibers (as exemplified by RC/MXene V) could compromise the mechanical performance since excessive nanofillers can form large aggregations, introducing defects within the matrix that facilitate crack initiation and premature fracture.

Additionally, the adequately porous structure within the RC/MXene composite cloth (RC/MXene IV) facilitates the outward transport of water vapor, achieving a water vapor transmission rate (WVTR) of 13.3 mg cm^−2^ h^−1^. This figure closely parallels the WVTR of commercially available cotton cloth (13.0 mg cm^−2^ h^−1^) (Fig. 3e), underscoring the essential breathability of RC/MXene cloth for human comfort.

Concurrently, we conducted water-wicking rate tests on RC/MXene IV, RC, CA, and cotton cloth. For each sample, a 5 × 5 cm^2^ segment absorbed 0.1 mL of deionized water on the platform, and the wicking rate was calculated by dividing the wicking area by the wicking time for each specimen [44]. Notably, owing to the interconnected fibrous and hydrophilic structures inherent in the RC/MXene IV cloth, the established water transport channels allow for rapid capillary action to swiftly draw water beneath the cloth and disperse it. This results in the highest wicking rate (30.7 ± 5.1 mm^2^ s^−1^) among the various samples (Fig. 3f). This design feature ensures a continuous and rapid water uptake capability, facilitating prompt evaporation.

According to the above-mentioned measurements, the combined capabilities encompass photo-to-thermal conversion, breathability, rapid water uptake, and durability coalesce within a single RC/MXene composite cloth. This convergence opens avenues for various applications, including personalized heating management and solar-driven water evaporation. Henceforth, this multifunctional amalgamation is referred to as the integrated functional cloth or, simply, *if*-Cloth.

### *if*-Cloth for Personal Heating Management

**Fig. 4:**
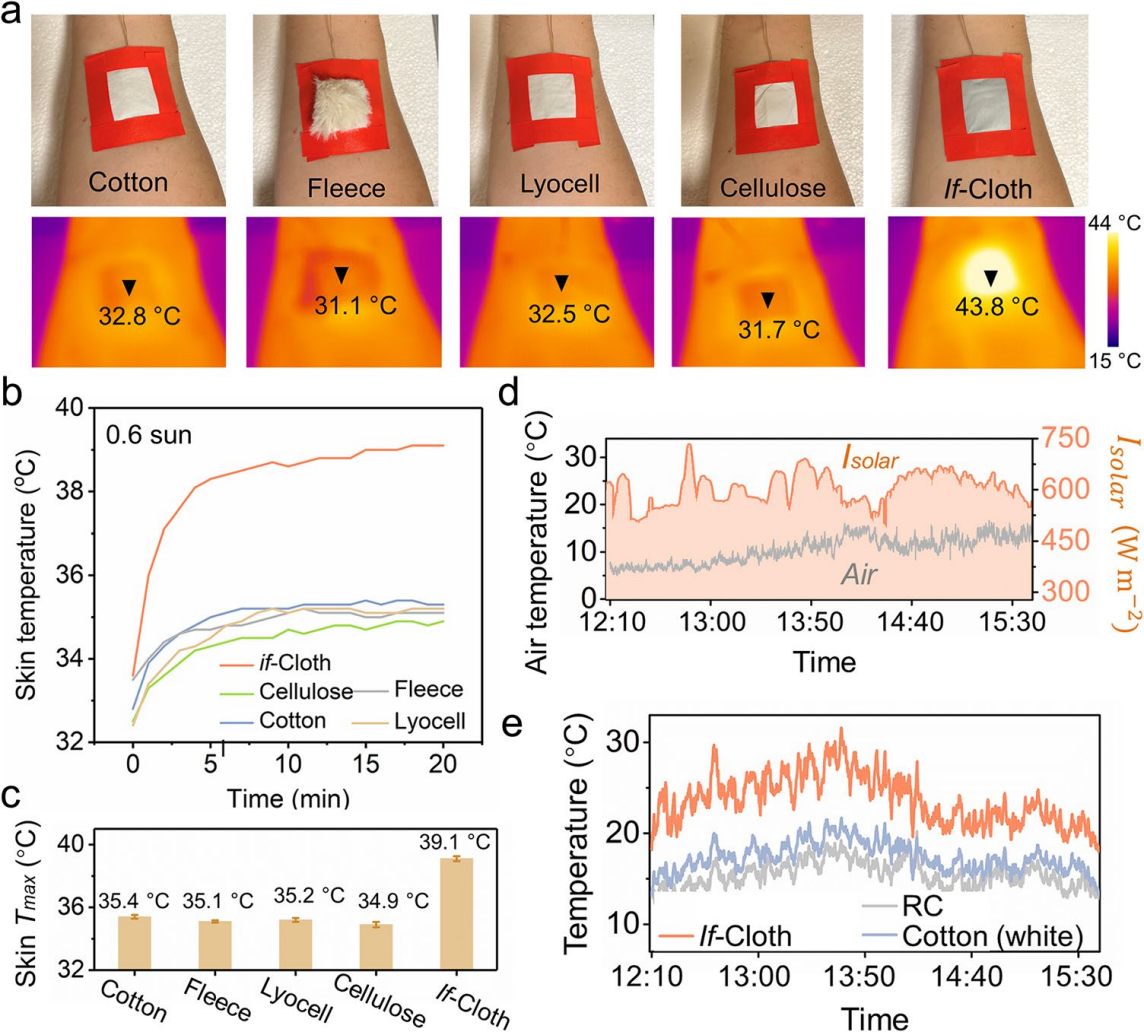
The *if*-Cloth for personal heating management. **a** The digital images of the arm skin covered by *if*-Cloth and other common fabrics as reference (cotton, fleece, lyocell and cellulose) of 3.5 × 4.0 cm^2^ in size and the corresponding thermal infrared images under 0.6-sun irradiation. **b** Time course plots of the temperature of human skin covered by *if*-Cloth and other common fabrics (cotton, fleece, lyocell and cellulose) under 0.6-sun irradiation and **c** their *T*_max_. **d** Detailed *I*_solar_ and *T*_ambient_ in Stockholm area (59° 22′ 13″ N, 18° 3′ 35″ E, November 12, 2022). **e** The real-time temperature of the simulated skin was covered by *if*-Cloth, RC nanofibers and a white cotton cloth over 3 h under sunlight in Stockholm, Sweden

Since *if*-Cloth can be employed to collect solar energy into a heat source to stand cold climates, it could potentially suit outer cover of the autumn/winter coats for warming human body in daytime. To evaluate heating performance in real-life, experiments were conducted on the skin of a human wrist covered individually by pieces of *if*-Cloth and other conventional fabrics (cotton, fleece, lyocell, and cellulose) of 3.5 × 4.0 cm^2^ in size as reference, as shown in Fig. 4a. As a result, *if*-Cloth (RC/MXene IV) registers a surface temperature increase to 43.8 °C after a 20-min exposure to 0.6-sun irradiation. This temperature surpasses that of cotton (32.8 °C), fleece (31.1 °C), lyocell (32.5 °C), and cellulose (31.7 °C) fabrics under identical conditions. Consequently, the heat from solar energy effectively transfers to the skin underneath *if*-Cloth (RC/MXene IV), leading to a skin temperature of 39.1 °C. Notably, *if*-Cloth's warming effect is superior to the other reference fabrics, which range from 34.9 to 35.4 °C (Fig. 4b, c).

Conventional textiles commonly retain body heat and inhibit its dissipation into the environment to keep the body warm [45, 46]. This approach involves insulating against thermal conduction and convection to maintain warmth. However, the efficacy of passive thermal radiation for heating is limited and struggles to adequately address complex outdoor conditions. In contrast, *if*-Cloth actively harnesses abundant outdoor solar radiation, converting it into heat to withstand colder climates. Consequently, garments constructed from *if*-Cloth can be envisioned as thinner and lighter (with a thickness of 0.1 mm and a weight of 30.1 g m^−2^) compared to conventional textiles, all while delivering superior warming capabilities for the human body.

To further showcase its remarkable solar heating prowess within cold climates, an outdoor assessment was conducted to gauge the solar heating capabilities of *if*-Cloth (RC/MXene IV), MXene-free RC nanofibers, and a white cotton cloth. This evaluation took place during autumn (on November 12, 2022) in Stockholm, characterized by an average ambient temperature (*T*_ambient_) of approximately 11.2 °C and an average solar intensity (*I*_solar_) of roughly 588 W m^−2^. Throughout 3 h, the experimental setup (Fig. S14) was exposed to sunshine, with the temperatures of a simulated skin surface covered by the respective samples being closely monitored. Concurrently, real-time data encompassing solar irradiance, ambient temperature, wind speed, and simulated skin temperature were meticulously recorded (Fig. 4d, e and Fig. S15).

The simulated skin enveloped by *if*-Cloth (RC/MXene IV) attains an average temperature (*T*_ave_) of around 21.3 °C. This is notably warmer than the ambient temperature (11.2 °C) by 10.1 °C, as well as the RC nanofibers (14.1 °C) and the cotton cloth (15.7 °C) by 7.2 °C and 5.6 °C, respectively. During the peak solar intensity of approximately 654 W m^−2^ (at 13:37 pm), *if*-Cloth achieves a maximum temperature (*T*_max_) of 31.6 °C. *If*-Cloth effectively imparts energy-efficient heating to a surface, tolerating daytime application scenarios in chilly environments.

### *if-*Cloth for Solar-Driven Water Evaporation

**Fig. 5:**
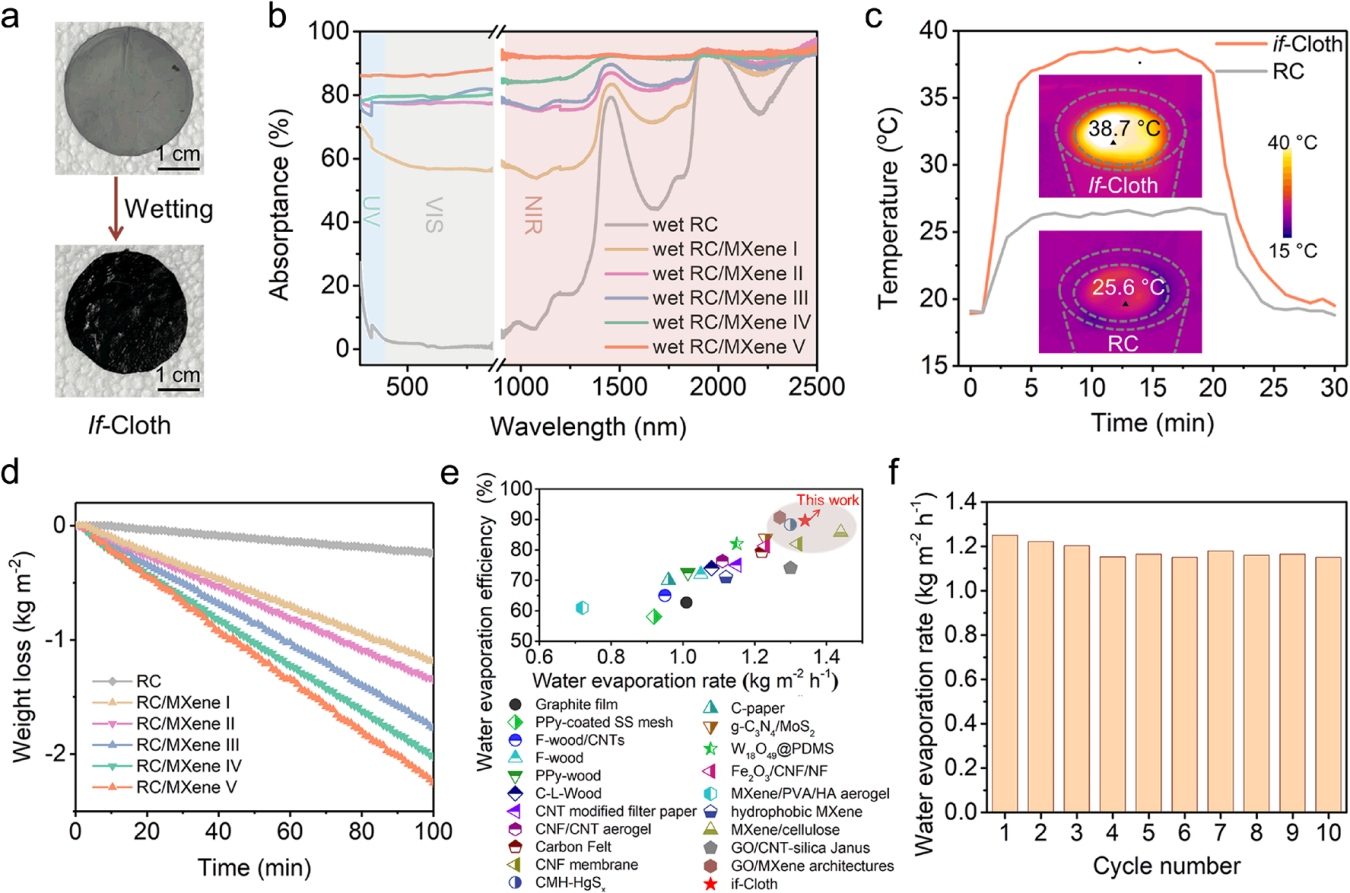
The *if*-Cloth for solar-driven water evaporation. **a** Optical images comparing the colors of *if*-Cloth in its dry and wet states. **b** UV–Vis-NIR absorption spectra of RC and RC/MXene I/II/III/IV/V nanofibers in their wet states. **c** Temperature profiles of *if*-Cloth and RC nanofibers wetted by pure water. Inset: IR images of *if*-Cloth (RC/MXene IV) and RC nanofibers under one-sun irradiation in equilibrium states. **d** Pure water evaporation kinetics of RC and RC/MXene I/II/III/IV/V nanofibers under one-sun irradiation. **e** Performance comparison of solar evaporators reported here and previously. **f** Synthetic seawater evaporation rates of *if*-Cloth (RC/MXene IV) in 10 repeated cycles under one-sun irradiation and rinsing by pure water

Impressively, when in a wet state, the *if*-Cloth displays a darker color compared to its dry state (Fig. 5a). Correspondingly, the wet *if*-Cloth (RC/MXene V) exhibits a significant increase in full light absorption (e.g., 87.7% of RC/MXene V in the 300–2500 nm range) compared to the dry state of *if*-Cloth (74.7%) (Fig. 5b, Fig. S16 and Table S6). In the wet *if*-Cloth, water acts as an intermediate medium between air and porous nanofibers, reducing light scattering at the water/cloth interface and thereby capturing more light (Supplementary Note S3) [27, 47]. Due to its excellent light harvesting properties, the wet *if*-Cloth accelerates the evaporation rate of water, leading to the rapid generation of water vapor in sunlight.

For the corresponding experiment, a round-shaped *if*-Cloth with a diameter of 1.6 cm was employed as a steam generator. It was positioned atop a 2.0 cm-thick polystyrene (PS) foam heat barrier to minimize heat dissipation from the *if*-Cloth to the underlying bulk water. To ensure complete wetting of the hydrophilic *if*-Cloth, a cellulose cloth strip was utilized as a capillary water channel to transport water upward to the *if*-Cloth. Real-time monitoring of temperature fluctuations and water mass loss was achieved using an infrared (IR) camera and a digital scale, respectively, as shown in Fig. S17.

As depicted in Fig. 5c, a wet *if*-Cloth (RC/MXene V) reached a maximum temperature (*T*_max_) of 38.7 °C within 10 min under one-sun irradiation. It then maintained this equilibrium state to continually facilitate water evaporation. In contrast, the wet MXene-free RC cloth reached a *T*_max_ of 25.6 °C under the same conditions, primarily due to its relatively lower photo-to-thermal conversion capability. By calculating the mass loss during pure water evaporation under one-sun irradiation, the water evaporation rates for RC and RC/MXene I/II/III/IV/V nanofibers were determined as 0.14 kg m^−2^ h^−1^, 0.70 kg m^−2^ h^−1^, 0.82 kg m^−2^ h^−1^, 1.02 kg m^−2^ h^−1^, 1.23 kg m^−2^ h^−1^, and 1.34 kg m^−2^ h^−1^, respectively (Fig. 5d). Notably, the incorporation of a higher concentration of MXene nanosheets into the nanofibers resulted in enhanced water evaporation rates. Consequently, the solar-to-water-evaporation efficiency was elevated, calculated at 9.5%, 47.5%, 55.7%, 68.2%, 82.3%, and 89.6%, respectively, for pure RC, RC/MXene I/II/II/IV/V nanofibers (Supplementary Note S4). Leveraging MXene nanosheets as photothermal additives [48–51], the solar-to-water-evaporation efficiency of *if*-Cloth (RC/MXene V) exceeded that of the MXene-free RC cloth by over 9 times. Compared with other artificial solar evaporators, *if*-Cloth demonstrated a superior capability for steam generation driven by sunlight (Fig. 5e and Table S7).

To further investigate the viability of *if*-Cloth for solar desalination, a synthetic seawater solution containing 3.5 wt% NaCl was prepared and subjected to continuous evaporation by *if*-Cloth under one-sun irradiation, utilizing the same measurement setup. As illustrated in Fig. S18, NaCl salts crystallized and accumulated on the surface of *if*-Cloth (RC/MXene IV) over a 24-h period of solar-driven synthetic seawater evaporation. Notably, the deposited salt could be easily cleaned by water (Movie S1). Consequently, *if*-Cloth maintained its performance, achieving a saltwater evaporation rate of 1.15–1.25 kg m^−2^ h^−1^ across sequential 10 cycles of testing (Fig. 5f and Fig. S19).

To provide a more comprehensive understanding of *if*-Cloth's real-life performance, a homemade solar desalination setup was employed to evaporate actual seawater (salinity: 3.6 g/kg). Within this configuration, a round-shaped *if*-Cloth (RC/MXene IV) measuring 1.6 cm in diameter was positioned within a plastic container and covered by a transparent plastic foil (Fig. S20). Exposed to typical solar irradiation (ranging from 350 to 850 W m^−2^) at *T*_ambient_ of approximately 15–24 °C, the evaporated water was collected and weighed, yielding a total of 8.98 kg m^−2^ over a single day (July 11–12, 2023). In summation, the durability of *if*-Cloth aptly suits the demands of practical solar desalination and salt removal applications.

## Conclusions

In summary, we have introduced a novel integrated functional cloth, *if*-Cloth, which is created by incorporating MXene nanosheets within regenerated cellulose nanofibers using a simple electrospinning technique. This *if*-Cloth (RC/MXene IV) exhibits strong photo-to-thermal conversion capability, rendering it effective as an outer layer for warming the human body in cold conditions. Equally significant, *if*-Cloth (RC/MXene V) is a valuable steam generator for clean water production, boasting an impressive water evaporation rate of 1.34 kg m^−2^ h^−1^ and a remarkable solar-to-water-evaporation efficiency of 89.6% under one-sun irradiation. Notably, *if*-Cloth employs a fossil-free energy-management approach, addressing the fundamental and critical human needs for water and warmth in an impressive synthesis of functionalities.

### Supplementary Information

Below is the link to the electronic supplementary material.

Supplementary file1 (DOCX 4420 KB)

Supplementary file2 (MP4 3723 KB)

## Data Availability

The data that support the findings of this study are available from the corresponding author upon reasonable request.
