# Supplementary material for: MXene/Cellulose Composite Cloth for Integrated Functions (if-Cloth) in Personal Heating and Steam Generation
Source: Adv Fiber Mater. 2023 Dec 22;6(1):252–63. doi: 10.1007/s42765-023-00345-w (PMC10943173; doi:10.1007/s42765-023-00345-w)
Supplement: Supplementary file 1 — Supplementary file1 (DOCX 4420 KB) [file 42765_2023_345_MOESM1_ESM.docx]

Supporting Information

**MXene/cellulose composite cloth for integrated functions (*if*-Cloth) in personal heating and steam generation**

Jian Chang,^a^ Bo Pang,^a^ Hao Zhang,^a^ Kanglei Pang,^a^ Miao Zhang,^a,*^ and Jiayin Yuan^a,*^

^a^ Department of Materials and Environmental Chemistry, Stockholm University, Stockholm, 10691, Sweden.

^*^ Corresponding author. E-mail address: miao.zhang@mmk.su.se (Miao Zhang); jiayin.yuan@mmk.su.se (Jiayin Yuan).





Fig. S1 UV-Vs-NIR reflection (a), absorption (b) and transmission (c) spectra of pure MXene film prepared *via* vacuum filtration of an aqueous MXene dispersion.





Fig. S2 The SEM images of (a) MAX powder and (b) MXene nanosheets.





Fig. S3 (a) SEM image of MXene nanosheets. (b) The size distribution plot of MXene nanosheets.





Fig. S4 (a) TEM image of MXene nanosheets. EDS mapping images of MXene nanosheet showing (b) C, (c) Ti, (d) O, and (e) F elements. (f) The corresponding element pattern of MXene nanosheet.





Fig. S5 (a) XPS spectrum of MXene nanosheets. (b) Fitted XPS spectra of Ti 2p of MXene nanosheets corresponding to Ti-C (455.2 and 461.0 eV), Ti-O (455.5 and 461.7 eV), and Ti-F (457.2 and 463.8 eV).





Fig. S6 (a) Tensile stress-strain curves of CA and RC nanofibrous membranes. (b) water contact angle measurement for CA and RC nanofibrous membranes. Optical (c) and SEM (d) images of the RC nanofibrous membrane exposed to 1 M HCl, 1 M NaOH, and chloroform solutions within one week.





Fig. S7 (a) Thermogravimetric analysis (TGA) curve of MXene film. (b) TGA curves of RC and RC/MXene I/II/III/IV/V nanofibers under N_2_ atmosphere at a heating rate of 10 °C min^-1^. (c) Derivative thermogravimetric analysis (DTG) curve representing thermal decomposition of RC and RC/MXene I/II/III/IV/V nanofibers.





Fig. S8 The diameter distribution plot of RC/MXene IV nanofibers.





Fig. S9 UV-Vis-NIR transmission spectra of RC and RC/MXene I/II/III/IV/V nanofibers of ~0.1 mm in thickness.





Fig. S10 UV-Vis-NIR reflection spectra of RC and RC/MXene I/II/III/IV/V nanofibers.





Fig. S11 Time course plot of the temperature of the pure MXene film under one-sun irradiation. Inset: the digital image of pure MXene film.





Fig. S12 Optical images of (a) pristine MXene film and (b) RC nanofibrous membrane (RC/MXene IV) under sonication for 20 min and then exposed to chloroform, 1 M HCl, and 1 M NaOH solutions within one month. The SEM images of RC/MXene IV membrane after treatment of (c) chloroform, (d) 1 M HCl and (e) 1 M NaOH solutions.





Fig. S13 FT-IR spectra of RC/MXene IV nanofibers exposed to chloroform, 1 M HCl, and 1 M NaOH solutions within one month.





Fig. S14 Digital image of the experimental setup for solar heating in an outdoor environment (Stockholm, 59°22'13" N, 18°3'35" E, 2022-11-12, 12:10 pm-15:10 pm).





Fig. S15 The real-time wind speed (Stockholm, 59°22'13" N, 18°3'35" E, 2022-11-12, 12:10 pm-15:10 pm).





Fig. S16 UV-Vis-NIR reflection spectra of RC and RC/MXene I/II/III/IV/V nanofibers after wetting by water.


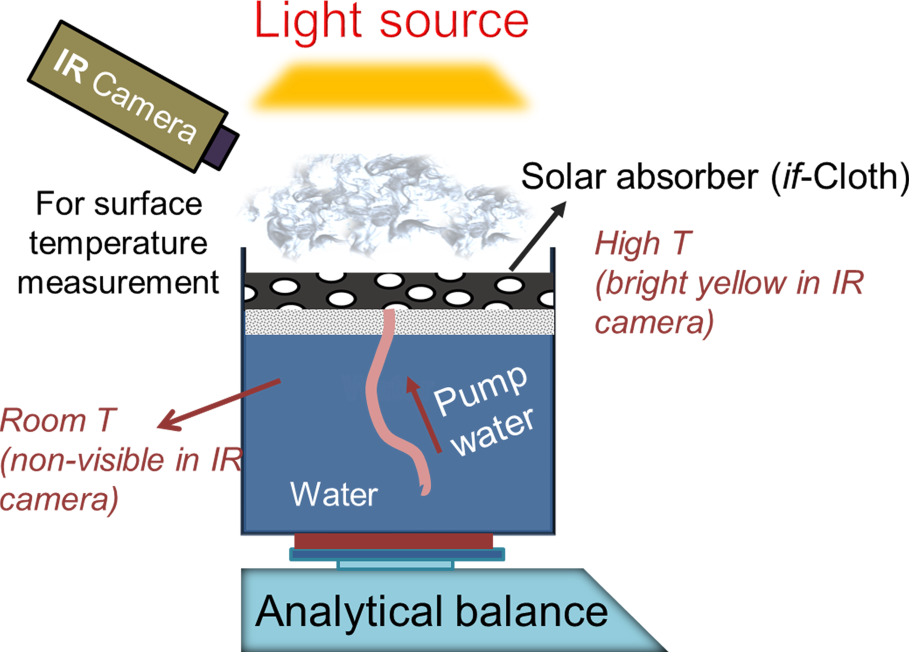


Fig. S17 Scheme of solar-driven water evaporation experimental setup.



Fig. S18 (a) The digital images of the deposition of NaCl salt on the surface of *if*-Cloth during 3 h, 6 h and 24 h one-sun irradiation. (b) The digital image of *if*-Cloth (RC/MXene IV) after rinsing by pure water.





Fig. S19 Salt water evaporation kinetics of *if*-Cloth (RC/MXene IV) under one-sun irradiation within 10 days.





Fig. S20 The home-made solar desalination setup. Inset: the bottles of seawater (left) and evaporated water (right).

Supplementary Note S1: calculation of MXene weight fraction in RC/MXene composite nanofibers (RC/MXene I/II/III/IV/V)

The TGA curves (Fig. S7) show mass losses in the temperature range between 280 °C and 600 °C. The first slight mass reduction below 280 °C is assigned to solvent evaporation, while major weight loss before 600 °C originates from the degradation of RC/MXene composite nanofibers. Herein, the MXene weight fraction in nanofibers is calculated at the range from 280 °C to 600 °C, and determined by following equation (1):

$w=\frac{M-M_{RC}}{M_{MXene}-M_{RC}}$ (1)

Where $w$ represents the MXene weight fraction in composite nanofibers; $M$, $M_{RC}$ and $M_{MXene}$ are the weight loss of composite nanofiber, regenerated cellulose (RC) nanofiber and MXene film between 280 °C and 600 °C, respectively. The detailed data for $M$, $M_{RC}$, $M_{MXene}$ and $w$ for RC/MXene composite nanofibers (RC/MXene I/II/III/IV/V) were listed in Table S2.

Supplementary Note S2: calculation of average spectral absorption of MXene film and RC/MXene composite nanofibers

The average spectral absorption (A) of sample at the range between $\lambda_{1}\mathrm{and}\lambda_{2}$is the irradiance-weighted average of its spectral absorption, which is calculated according to the following equation (2) [1]:

$A=\frac{\int_{\lambda_{1}}^{\lambda_{2}} \alpha(\lambda)i(\lambda)d(\lambda)}{\int_{\lambda_{1}}^{\lambda_{2}} i(\lambda)d(\lambda)}$ (2)

Where $\alpha$(𝜆) is spectral absorption, $\alpha=1-r-$*t* ($r$ and *t* are spectral reflection and transmission, respectively). The spectral reflection ($r$) and transmission (*t*) were measured by using UV-Vis-NIR spectrometer accompanied by an integrating sphere attachment. $i\left( \lambda\right)$ is the solar spectral irradiance (W m^-2^ nm^-1^), obtained from ASTM standard G173-03. As indicated in Fig. S9, there is no light transmission through these nonwoven nanofibers and thus the light reflection and adsorption add up to unity (100%). Herein, the range of visible light is between $\lambda_{1}$~400 nm and $\lambda_{2}$~700 nm; the range of NIR light is between $\lambda_{1}$~700 nm and $\lambda_{2}$~2500 nm; the range of solar light is between $\lambda_{1}$~300 nm and $\lambda_{2}$~2500 nm.

Supplementary Note S3: reflectivity in different media

Light possesses different velocities as it travels in different media. That can be characterized by the specific refractive index (n) for the light and the medium. The refractive indices of air, water and cellulose (in this work) are 1.00, 1.33, and 1.47, respectively [2]. According to the Fresnel equation [3], the reflectance of light at the air/water interface (*R*_air/water_), air/cellulose interface (*R*_air/cellulose_) and water/cellulose interface (*R*_water/cellulose_) can be calculated as equation (3):

$R=\left( \frac{n_{1}-n_{2}}{n_{1}+n_{2}} \right)^{2}$ (3)

Where $n_{1}$ and $n_{2}$ are the refractive indices for medium 1 and medium 2. Therefore, the *R*_air/water_, *R*_air/cellulose_ and *R*_water/cellulose_ are 2.0%, 3.6% and 0.25%, respectively. It is noted that the calculation does not consider the incident light angle effect and they are only rough estimations for further discussion. According to equation (3), it is indicated that adding an intermediate layer (water) whose refractive index is between medium 1 (air) and 2 (cellulose) would reduce the total refection loss. Therefore, the water layer acting as the antireflective layer, decreases the reflection of *if*-Cloth.

Supplementary Note S4: calculation of the solar-to-water-evaporation efficiency of nanofibers

The solar-to-water-evaporation efficiency (η) of the photothermal nanofibers is calculated using equation (4) [4]:

$\eta=\frac{Q_{e}}{Q_{s}}$ (4)

Where $Q_{s}$ is the power density of solar irradiation (1000 W m^−2^) and $Q_{e}$ is the power for water evaporation, which is further defined by equation (5):

$Q=\frac{dm\times H_{e}}{dt}=v\times H_{e}$ (5)

Where m is the mass of evaporated water, t is time, $H_{e}$ is the latent heat of water evaporation (2444 kJ kg^−1^ at 25 °C and 2408 kJ kg^−1^ at 40 °C, respectively) and $v$ is the water evaporation rate, which is calculated by using equation (6):

$v=\frac{dm}{S\times dt}$ (6)

Where m is the mass of evaporated water, S is the surface area of the photothermal nanofibers, and t is the duration time.

Table S1. Atomic fraction of elements (C, O, F and Ti) in MXene nanoflakes obtained from EDS analysis.

| Elements | Atomic Fraction (%) | Atomic Error (%) |
| --- | --- | --- |
| C | 27.86 | 4.13 |
| O | 28.62 | 7.15 |
| F | 10.55 | 2.63 |
| Ti | 32.97 | 6.56 |

Table S2 The weight loss of MXene nanosheets, RC, and RC/MXene composite (RC/MXene I/II/III/IV/V) nanofibers between 280 °C and 600 °C.

| Sample | Weight loss between 280 °C  and 600 °C (wt%) | Input MXene content (wt%) | MXene content by TGA (wt%) |
| --- | --- | --- | --- |
| MXene | 7.26 | - | - |
| RC | 85.28 | - | - |
| RC/MXene I | 84.30 | 1 | 1.3 |
| RC/MXene II | 83.82 | 3 | 1.9 |
| RC/MXene III | 82.68 | 5 | 3.3 |
| RC/MXene IV | 80.84 | 7 | 5.7 |
| RC/MXene V | 79.03 | 9 | 8.0 |

Table S3 The diameter distribution of RC/MXene IV nanofibers.

| Distribution (μm) | Mean diameter (μm) | Frequency (%) |
| --- | --- | --- |
| 0-0.1 | 0.05 | 0 |
| 0.1-0.2 | 0.15 | 18 |
| 0.2-0.3 | 0.25 | 60 |
| 0.3-0.4 | 0.35 | 16 |
| 0.4-0.5 | 0.45 | 6 |
| 0.5-0.6 | 0.55 | 0 |

Table S4 Solar spectra (300-2,500 nm) absorption of MXene film, and RC and RC/MXene composite (RC/MXene I/II/III/IV/V) nanofibers, and their maximum temperatures (*T*_max_) under one-sun irradiation.

| Sample | Solar absorption (%)  (300-2,500 nm) | *T*_max_ (°C) |
| --- | --- | --- |
| MXene | 68.64 | 62.6 |
| RC | 12.67 | 34.9 |
| RC/MXene I | 35.46 | 45.9 |
| RC/MXene II | 53.36 | 50.5 |
| RC/MXene III | 59.17 | 52.2 |
| RC/MXene IV | 63.35 | 55.4 |
| RC/MXene V | 74.67 | 62.1 |

Table S5 The mechanical property data of CA, RC and RC/MXene composite (RC/MXene I/II/III/IV/V) nanofibers.

| Sample | Strength (MPa) | Strain (%) | Toughness (MJ/m^3^) | Modulus (MPa) |
| --- | --- | --- | --- | --- |
| CA | 0.37 ± 0.01 | 12.04 ± 1.13 | 0.02 ± 0.01 | 12.25 ± 5.29 |
| RC | 1.52 ± 0.26 | 12.20 ± 0.93 | 0.08 ± 0.04 | 95.26 ± 13.84 |
| RC/MXene I | 3.94 ± 0.95 | 9.72 ± 1.36 | 0.18 ± 0.04 | 229.88 ± 39.87 |
| RC/MXene II | 4.76 ± 1.09 | 6.76 ± 0.96 | 0.11 ± 0.04 | 255.83 ± 69.29 |
| RC/MXene III | 5.61 ± 0.13 | 11.27 ± 0.95 | 0.15 ± 0.02 | 398.13 ± 161.75 |
| RC/MXene IV | 8.63± 1.96 | 11.04 ± 3.87 | 0.48 ± 0.30 | 453.54 ± 125.48 |
| RC/MXene V | 2.92 ± 0.46 | 6.51 ± 3.81 | 0.05 ± 0.02 | 225.08 ± 57.06 |

Table S6 Solar spectra (300-2,500 nm) absorption of RC and RC/MXene composite (RC/MXene I/II/III/IV/V) nanofibers after wetting by water.

| Sample | Solar absorption (%)  (300-2,500 nm) |
| --- | --- |
| RC (wet) | 9.93 |
| RC/MXene I (wet) | 59.29 |
| RC/MXene II (wet) | 74.47 |
| RC/MXene III (wet) | 78.94 |
| RC/MXene IV (wet) | 81.42 |
| RC/MXene V (wet) | 87.72 |

Table S7 The water evaporation rate and solar-to-water evaporation efficiency of solar evaporators reported previously and our *if*-Cloth.

| **Materials** | **Evaporation rate (kg·m^-2^·h^-1^)** | **Efficiency (%)** | **Ref.** |
| --- | --- | --- | --- |
| PPy-coated SS mesh | 0.92 | 58 | [4] |
| Graphite film | 1.01 | 62.7 | [5] |
| F-wood/CNTs | 0.95 | 65 | [6] |
| F-wood | 1.05 | 72 | [7] |
| PPy-wood | 1.01 | 72.5 | [8] |
| C-L-Wood | 1.08 | 74 | [9] |
| CNT modified filter paper | 1.15 | 75 | [10] |
| CNF/CNT aerogel | 1.11 | 76.3 | [11] |
| Carbon Felt | 1.22 | 79.4 | [12] |
| CNF membrane | 1.32 | 82 | [13] |
| CMH-HgS_x_ | 1.30 | 88.3 | [14] |
| C-paper | 0.96 | 70 | [15] |
| g-C_3_N_4_/MoS_2_ | 1.23 | 83.8 | [16] |
| W_18_O_49_@PDMS | 1.15 | 82.0 | [17] |
| Fe_2_O_3_/CNT/NF | 1.23 | 81.3 | [18] |
| MXene/PVA/HA aerogel | 0.72 | 61.0 | [19] |
| hydrophobic MXene | 1.12 | 71.0 | [20] |
| MXene/cellulose | 1.44 | 85.8 | [21] |
| GO/CNT-silica Janus | 1.30 | 74 | [22] |
| GO/MXene architectures | 1.27 | 90.7 | [23] |
| *if*-Cloth | 1.34 | 89.6 | This work |

**Reference**

[1] Shi M, Shen M, Guo X, Jin X, Cao Y, Yang Y, Wang W, Wang J. Ti_3_C_2_T_x_ MXene-decorated nanoporous polyethylene textile for passive and active personal precision heating. *ACS Nano* **2021**, *15*: 11396.

[2] Jin Y, Chang J, Shi Y, Shi L, Hong S, Wang P. A highly flexible and washable nonwoven photothermal cloth for efficient and practical solar steam generation. *J. Mater. Chem. A* **2018**, *6*: 7942.

[3] Hapke B. Theory of reflectance and emittance spectroscopy. 2nd ed. Cambridge university press, **2012**.

[4] Zhang L, Tang B, Wu J, Li R, Wang P. Hydrophobic light-to-heat conversion membranes with self‐healing ability for interfacial solar heating. *Adv. Mater.* **2015**, *27*: 4889.

[5] Kashyap V, Al-Bayati A, Sajadi SM, Irajizad P, Wang SH, Ghasemi H. A flexible anti-clogging graphite film for scalable solar desalination by heat localization. *J. Mater. Chem. A* **2017**, *5*: 15227.

[6] Chen C, Li Y, Song J, Yang Z, Kuang Y, Hitz E, Jia C, Gong A, Jiang F, Zhu JY, Yang B, Xie J, Hu L. Highly flexible and efficient solar steam generation device. *Adv. Mater.* **2017**, *29*: 1701756.

[7] Xue G, Liu K, Chen Q, Yang P, Li J, Ding T, Duan J, Qi B, Zhou J. Robust and low-cost flame-treated wood for high-performance solar steam generation. *ACS Appl. Mater. Interfaces* **2017**, *9*: 15052.

[8] Wang Z, Yan Y, Shen X, Jin C, Sun Q, Li H. A wood-polypyrrole composite as a photothermal conversion device for solar evaporation enhancement. *J. Mater. Chem. A* **2019**, *7*: 20706.

[9] Liu H, Chen C, Chen G, Kuang Y, Zhao X, Song J, Jia C, Xu X, Hitz E, Xie H, Wang S, Jiang F, Li T, Li Y, Gong A, Yang R, Das S, Hu L. High‐performance solar steam device with layered channels: artificial tree with a reversed design. *Adv. Energy Mater.* **2018**, *8*: 1701616.

[10] Yang P, Liu K, Chen Q, Li J, Duan J, Xue G, Xu Z, Xie W, Zhou J. Solar-driven simultaneous steam production and electricity generation from salinity. *Energy Environ. Sci*. **2017**, *10*: 1923.

[11] Jiang F, Liu H, Li Y, Kuang Y, Xu X, Chen C, Huang H, Jia C, Zhao X, Hitz E, Zhou Y, Yang R, Cui L, Hu L. Lightweight, mesoporous, and highly absorptive all-nanofiber aerogel for efficient solar steam generation. *ACS Appl. Mater. Interfaces* **2018**, *10*: 1104.

[12] Li H, He Y, Hu Y, Wang X. Commercially available activated carbon fiber felt enables efficient solar steam generation. *ACS Appl. Mater. Interfaces* **2018**, *10*: 9362.

[13] Wang Y, Zhang L, Wang P. Self-floating carbon nanotube membrane on macroporous silica substrate for highly efficient solar-driven interfacial water evaporation. *ACS Sustain. Chem. Eng*. **2016**, *4*: 1223.

[14] Li M, Yang M, Liu B, Guo H, Wang H, Li X, Wang L, James TD. Self-assembling fluorescent hydrogel for highly efficient water purification and photothermal conversion. *Chem. Eng. J.* **2022**, *431*: 134245.

[15] Liu S, Huang C, Luo X, Rao Z. High-performance solar steam generation of a paper-based carbon particle system. *Appl. Therm. Eng*. **2018**, *142*: 566.

[16] Gan Q, Xiao Y, Li C, Peng H, Zhang T, Ye M. g-C_3_N_4_/MoS_2_ based floating solar still for clean water production by thermal/light activation of persulfate. *Chemosphere* **2021**, *280*: 130618.

[17] Chang Y, Wang Z, Shi Y-e, Ma X, Ma L, Zhang Y, Zhan J. Hydrophobic W_18_O_49_ mesocrystal on hydrophilic PTFE membrane as an efficient solar steam generation device under one sun. *J. Mater. Chem. A* **2018**, *6*: 10939.

[18] Han S, Yang J, Li X, Li W, Zhang X, Koratkar N, Yu ZZ. Flame synthesis of superhydrophilic carbon nanotubes/Ni foam decorated with Fe_2_O_3_ nanoparticles for water purification *via* solar steam generation. *ACS Appl. Mater. Interfaces* **2020**, *12*: 13229.

[19] Cao S, Wu X, Zhu Y, Gupta P, Martinez A, Zhang Y, Ghim D, Wang Y, Liu L, Jun YS, Singamaneni S. MXene aerogel for efficient photothermally driven membrane distillation with dual-mode antimicrobial capability. *J. Mater. Chem. A* **2021**, *9*: 22585.

[20] Zhao J, Yang Y, Yang C, Tian Y, Han Y, Liu J, Yin X, Que W. A hydrophobic surface enabled salt-blocking 2D Ti_3_C_2_ MXene membrane for efficient and stable solar desalination. *J. Mater. Chem. A*. **2018**, *6*: 16196.

[21] Zha XJ, Zhao X, Pu JH, Tang LS, Ke K, Bao RY, Bai L, Liu ZY, Yang MB, Yang W. Flexible anti-biofouling MXene/cellulose fibrous membrane for sustainable solar-driven water purification. *ACS Appl. Mater. Interfaces* **2019**, *11*: 36589.

[22] Li L, Zang L, Zhang S, Dou T, Han X, Zhao D, Zhang Y, Sun L, Zhang Y. GO/CNT-silica Janus nanofibrous membrane for solar-driven interfacial steam generation and desalination. *J. Taiwan Inst. Chem. E.* **2020**, *111*: 191.

[23] Ming X, Guo A, Zhang Q, Guo Z, Yu F, Hou B, Wang Y, Homewood KP, Wang X. 3D macroscopic graphene oxide/MXene architectures for multifunctional water purification. *Carbon* **2020**, *167*: 285.
